# Communication board in locked-in syndrome: a practical interaction method with the patient

**DOI:** 10.1590/1980-5764-DN-2023-0041

**Published:** 2023-11-10

**Authors:** Gabriel de Deus Vieira, Zenóbio Cosme Gonçalves Ferreira, Lucas Nóbrega, Francisco Saulo Sampaio Cardoso, Eduardo Martins Leal, Rachel Schlindwein

**Affiliations:** 1 Universidade Federal de Santa Catarina, Departamento de Neurologia, Florianópolis SC, Brazil.; 2 Universidade Federal de Santa Catarina, Departamento de Neuropsiquiatria, Florianópolis SC, Brazil.

**Keywords:** Locked-in Syndrome, Communication Disorders, Nonverbal Communication, Síndrome do Encarceramento, Transtornos da Comunicação, Comunicação não Verbal

## Abstract

Locked-in syndrome is a neurological condition characterized by tetraplegia, mutism, preservation of vertical eye movement, superior eyelid movement, and intact consciousness, making it impossible for the patient to communicate properly. We herein describe a case to analyze the practice of developing a method of communication for a patient with locked-in syndrome. Two communication boards were created, adapted to the Portuguese language, as well as a shortcut to inquire about the physical and emotional patient’s well-being. We had difficulty with the initial communication board, due to the patient’s low education level, so we adapted a new one to the patient’s social context, including a shortcut to inquire about physical and emotional well-being. The communication board had a positive impact on treatment development and the patient’s life.

## RESUMO

A síndrome do encarceramento é uma condição neurológica caracterizada por tetraplegia, mutismo, preservação do movimento vertical dos olhos, movimento palpebral superior e consciência intacta, impossibilitando a comunicação adequada do paciente. Descrevemos um caso para analisar a prática de criação de um método de comunicação em um paciente com síndrome do encarceramento. Foram criadas duas pranchas de comunicação, adaptadas à língua portuguesa, e um atalho para indagar sobre o bem-estar físico e emocional do paciente. Tivemos dificuldade com a prancha de comunicação inicial em razão da baixa escolaridade do paciente, então criamos uma nova prancha de comunicação adaptada ao seu contexto social. A nova prancha tinha um atalho para indagar sobre o bem-estar físico e emocional do paciente. A prancha de comunicação teve impacto positivo no desenvolvimento do tratamento e na vida do paciente.

## INTRODUCTION

Locked-in syndrome (LIS) is a neurological condition characterized by tetraplegia, mutism, preservation of vertical eye movement, superior eyelid movement, and intact consciousness^1-3^. Patients inflicated with this syndrome are awake and conscious, but in a partial way, in other words, they are unable to make face or body movements or speak^4,5^.

There is a lack of efficient communication strategies with the family and healthcare professionals, impairing treatment, accessibility, and quality of life. In this way, this article aims to present and analyze the practice of the neurology and neuropsychology team on a patient with LIS.

## CASE REPORT

A 43- year-old female with 3^rd^ grade of Brazilian basic education (equivalent to the 3^rd^ grade in the United States), who worked part-time in a recycling company, was transferred to the neurology team with LIS.

In this case report, we used a communication system with the patient — a board was adapted to the Portuguese language and a shortcut was created to inquire about physical and emotional well-being. Our first concern was to establish a way of communicating. We identified an alphabetical board and communication system that could be easier for the patient to communicate “yes” and “no” through vertical eye movement. The patient, family, and medical team agreed that looking up meant “yes” and looking down meant “no”, conditioning these commands. Repetition training was done with the patient to learn the code. In this training, we made questions that we already knew the answers to in order to ensure that the patient understood and performed as expected.

**Chart 1.**
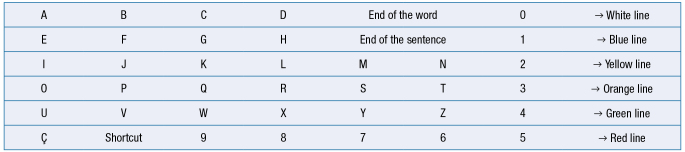
Communication board adapted with alphabet, numbers, and shortcuts for Brazilian Portuguese.

The communication board (Chart 1) was developed based on the alphabetical board by Khanna et al.^6^, and adapted to this context. Besides the alphabet and the terms “end of the word“ and “end of the phrase“, we added: *cedilla* (ç) to help in the Brazilian Portuguese language; the numbers from 0 to 9, and the possibility to turn the plate indicating the word “shortcut“.

We observed that, besides the regular alphabetical order, the board’s layout presented the vowels in vertical sequence, helping words formation. The same applied to the numbers, which were arranged in sequence from top to bottom and from right to left. Theoretically, with the question “Is the next letter in the blue/white/yellow/orange/green line?” the patient should be able to select the desired line by looking up when she heard the desired color. Then, the pencil was slowly passed over each letter on the line, observing if the patient followed it, if she attempted to select a letter, and so on for each line. Also, after using a consonant, it was possible to make the process faster by asking “Is the next letter a vowel?”, and when positive, moving the pencil vertically: A, E, I, O, U”. However, the patient in this study needed to see the word that the professional was writing so she did not lose her line of thought (written communication and reading were probably not present in the patient’s life and she did not consolidate this ability). For that reason, the professional held a notebook below the board with the selected letters written down. Besides, before the professional began a new inquiry, they gave her some time to think and to select the next letter she wanted to use.

**Figure 1.**
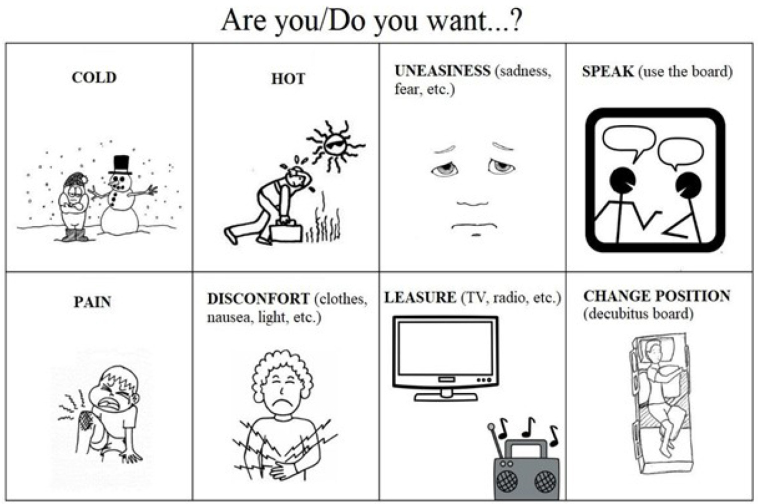
Shortcut of the communication board of Chart 1, for fast inquiry about physical and emotional well-being, created by the neuropsychology team.

There was another board called “shortcut” on the back of this board (Figure 1). On that side, some emotions and sensations references were presented in order to quickly trace the current comfort and well-being of the patient. Based on the board communication, it was identified that the frequent crying of the patient was more related to anxiety instead of a depressed mood. Therefore, some measures were taken so she felt more in control of her environment and could deal with her emotions in crisis moments: a clock was placed inside the patient’s field of vision; she was informed about the hospital’s routine; and an agenda was made so that she knew when visits would occur and when to wait for each professional, also to guide the family and the health team. Thus, feeling herself more supported and assisted, the patient established schedules for watching specific programs on TV and for playful activities. After the implementation of these strategies, there was an improvement in the patient’s anxiogenic symptoms and a reduction in the crying frequency, which did not completely cease, but is something understandable seeing the emotional vulnerability due to her clinical state and the long hospitalization period she lived.

This study was conducted in the neurology ward at the hospital of the Federal University of Santa Catarina, Florianópolis, Brazil. The CAAE number is 00783512.2.0000.0121.

## DISCUSSION

Communication with a patient with LIS must be one of the most important objectives of the medical team^7^. Bauby^8^ speaks of his personal experience as a person with LIS and of the solitude in his days in the hospital, where only two of the health professionals used the communication board with him. This communication helps express basic issues such as cold and even pain. It can point out a worsening of the patient’s clinical state or the appearance of secondary illnesses. Communication with the board is slow and requires patience, but it is the only accessibility resource that some patients have^9^.

Currently, there are some sophisticated forms of communication with these patients, such as brain interface devices and computers adapted to surf the Internet; however, they are not accessible to all patients, especially those with low financial conditions^9^. So, the communication board is a cheap and practical way to communicate, depending only on the patient’s look and grammar knowledge. Kopsky et al.^10^ also developed a communication board similar to this one, improving their patient’s communication up to three times faster than the existing spelling systems, which showed the effectiveness of this method in this kind of patient.

In conclusion, LIS is a rare neurological disorder that usually results from a pontine or extrapontine injury, while preserving conscience and relatively intact cognitive functions, associated with important motor capacity and verbal communication restrictions. In this context, communication, neuropsychological and psychotherapeutic evaluation, and intervention are complex. Knowing that this syndrome is extremely disabling for daily life activities, the intervention of a multidisciplinary team from several areas of knowledge is fundamental. This board enables, in a simple and practical way, an effective communication with patients with LIS and similar diseases, facilitating and improving their quality of life.
